# Assessing the impact of educational intervention based on a health belief model to modify cardiovascular disease risk factors among Egyptian University administrative staff: a quasi-experimental study

**DOI:** 10.1186/s12872-025-05359-3

**Published:** 2025-12-05

**Authors:** Abeer H. Ahmed, Nashwa I. Basyoni, Maha M. Wahdan, Nanees A. Ismail, Hoda I. Fahim

**Affiliations:** Department of Community, Environmental and Occupational Medicine. Faculty of Medicine, Ain Shams University, 38 Ramses Street, Abbasia Square, Cairo, 11566 Egypt

**Keywords:** Behavior change, Cardiovascular diseases, Health Education, Lifestyle, Risk factors

## Abstract

**Background:**

Globally, cardiovascular diseases (CVDs) are acknowledged as a primary cause of death and morbidity. Using the health belief model (HBM) can help individuals believe in adopting a healthy lifestyle and lower their modifiable risk of developing CVDs.

**Aim:**

The study was conducted to assess the effect of an educational program on changing CVDs health beliefs and motives among administrative staff at the faculty of medicine at Ain Shams University (ASU).

**Methods:**

A quasi-experimental study was conducted at the Faculty of Medicine at ASU. CVDs risk assessment was done for 214 administrative staff, and 79 of them agreed to participate in the interventional stage. Data were collected before the educational sessions and at 3-, 6-, and 9-month intervals after the sessions.

**Results:**

The study included 214 participants, mostly females (74.3%), with a mean age of 48.9 ± 7.95 years old. Based on the WHO/ISH prediction risk score, 46.7% of administrative staff had low CVDs risk, 31.3% had moderate risk, and 22% had high risk. Males mainly had a moderate risk (50.9%) and high risk (27.3%) of developing CVDs. Among participants enrolled in the educational program (79), the mean total score of the HBM subscales revealed significant improvements from baseline to the 3-month follow-up, except for the preventive behavior subscale. However, there was a slight decline at the 6- and 9-month follow-up periods. At 9 months, significant improvements were observed in most HBM constructs, including perceived susceptibility, severity, benefits, barriers, cues to action, and self-efficacy (effect sizes = 0.130–0.312, all *p* = 0.001). The total health score also increased (effect size = 0.322, *p* = 0.001). In contrast, preventive behavior showed no meaningful change (effect size = 0.010, *p* = 0.390).

**Conclusions:**

According to the WHO/ISH risk score, more than half of the individuals had a moderate to high risk of CVDs. The study demonstrated that administrative staff members' knowledge of CVDs behavioral risk factors was successfully increased by a brief HBM-based educational program. All HBM domain scores showed a significant increase, except for “preventive behavior” domain score, after three months; however, after six and nine months, there was a small decline.

**Supplementary Information:**

The online version contains supplementary material available at 10.1186/s12872-025-05359-3.

## Introduction

Cardiovascular diseases (CVDs) have long been recognized as a major cause of mortality and morbidity around the world [1]. These include disorders such as hypertension, coronary heart disease, atherosclerosis, and stroke that involve pathologies of the heart and blood vessels [2]. CVDs are considered one of the leading three causes of deaths in both developed and developing countries [3].

CVDs not only cause significant morbidity and mortality but also impose substantial social and economic burdens. By 2030 more than 22.2 million people will die annually from CVDs. Populations in low and middle-income countries (LMICs) now contribute to 75% of the CVDs deaths, which leads to 7% reduction of gross domestic product (GDP) in these countries [4].

Furthermore, CVDs are responsible for 85% of all disabilities worldwide and require long-term treatment, which can lead to financial hardship, especially among families in LMICs [4]. The situation is particularly severe in Africa, where CVDs are expected to account for over 50% of all deaths by 2030 [5]. Due to weak health infrastructure dual burden of disease and a severe lack of knowledge about CVDs risk factors in developing countries, these nations have demonstrated an inability to cope with the rising incidence of CVDs [6].

In Egypt, CVDs have been the leading cause of premature death since the 1990s. The World Health Organization (WHO) reports that 32.4% of all deaths in Egypt are due to coronary heart disease, placing the nation 15th in the world for coronary heart disease-related mortality [7].

There are several risk factors for CVDs that are generally divided into two categories: non-modifiable (age, sex, race, and family history) and modifiable (diabetes, lipid profile, hypertension, alcohol consumption, smoking, inadequate physical activity, inappropriate diet, and obesity). Behavioral, environmental, and social factors are the other risk factors for CVDs [8]. Global evidence indicates that by controlling and managing the modifiable risk factors, up to 90% of cases of CVDs can be reduced [9]. Thus, identifying persons more at risk of developing CVDs is of utmost importance.

Several risk prediction methods for different CVDs are available including the Framingham Risk Score (FRS), the American College of Cardiology/American Heart Association (ACC/AHA), Q RESEARCH cardiovascular risk algorithm (QRISK), Systematic Coronary Risk Evaluation (SCORE), Pooled Cohort Equation, and the (WHO)/ISH chart [10].

Of these, the WHO/ISH chart is easily applied and simple to calculate. The WHO/ISH model assesses the 10-year overall risk of cardiovascular illnesses for individuals between the ages of 40 and 79 in 14 regions. Both laboratory-based and non-laboratory-based sets of charts are available. Age, sex, smoking status, systolic blood pressure, and body mass index (BMI) are used for stratification in non-laboratory-based charts [11].

Furthermore, understanding and modifying behaviors that contribute to CVDs risk is essential. Research indicates that using behavior change theories and models is necessary to comprehend these elements [12]. Commonly used frameworks include the Health Belief Model (HBM), Transtheoretical Model, Theory of Planned Behavior (TPB), and Social Cognitive Theory (SCT), each offering unique insights based on factors like risk perception, readiness to change, social influence, and self-efficacy [13].

Compared with other behavior change frameworks, such as the Theory of Planned Behavior (TPB) -which emphasizes intention shaped by attitudes, subjective norms, and perceived behavioral control—or the Social Cognitive Theory (SCT)—which highlights reciprocal determinism, observational learning, and self-efficacy—the HBM offers a more direct focus on the individual's risk perception and cost–benefit appraisal. The HBM is more appropriate for situations where the target population (administrative staff) may not be fully aware of their CVDs risk and where the intervention aims to heighten personal risk awareness, address perceived barriers, and reinforce the benefits of preventive behaviors [14].

The HBM is particularly effective because it focuses on individual beliefs—such as perceived susceptibility, perceived severity, perceived benefits, and perceived barriers—which are directly relevant to promoting healthy behaviors and preventing non-communicable diseases like CVDs. The HBM is widely accepted for its strong predictive ability and structured framework for exploring why individuals adopt health behaviors [15]. It has been proven especially beneficial in public health areas such as smoking cessation, breast self-exams, nutrition, and cardiovascular disease prevention by targeting personal beliefs and motivations to support behavioral change [16].

Administrative staff are expected to be at higher risk for developing CVDs. This is because of their age distribution, sedentary lifestyle, prolonged hours of sitting at desks and intermediate socioeconomic status, all of which are significant risk factors for CVDs. Also, interaction with the public and carrying the responsibility of solving their problems is a substantial source of stress [17]. Thus, the Faculty of Medicine administrative staff is a subset of administrative staff who are more susceptible to CVDs than the general population, similar to administrative staff from other faculties, and they were chosen for this research.

After reviewing the literature, we found that prior work based on educational interventions in comparable settings have mostly concentrated on short-term results (3month follow up) or one-component initiatives, either screening or health education. Our intervention integrated screening and periodic monitoring. In contrast to other studies, we evaluated our participants at three, six, and nine months. The extended 9-month follow-up enabled us to evaluate the sustainability of behavioral changes after the first intervention period, which makes our study unique.

Thus, to address this issue of CVDs risk, the objective of the current study was to assess 10-year CVDs risk patterns among administrative staff at the Faculty of Medicine at Ain Shams University (ASU) using the WHO/ISH charts and to evaluate the effect of a health education program based on the HBM in changing their CVDs risk health beliefs and motives. By doing this our ultimate aim is to aid in identifying areas prone to improvement and corrective actions in order to decrease the CVDs risk in our community and the global community as well.

### Methodology

#### Setting

Faculty of Medicine, Ain Shams University (ASU).

#### Participants

Administrative staff 's ASU faculty of medicine.

- Inclusion criteria: Screening for CVDs focused on administrative staff who were 40 years and above.
- Exclusion criteria: These participants were excluded if they had a history of myocardial infarction, stroke, coronary artery disease, or any cardiovascular surgeries.

#### Study design

This study was conducted in two phases. Phase I comprised a cross-sectional assessment of CVDs risk among the administrative staff using the WHO risk prediction chart for non-laboratory-based CVDs (screening for cardiovascular disease risk). Phase II comprised an educational intervention based on the HBM which aimed at amending CVD's health beliefs and motives among the administrative staff. A quasi-experimental design was applied, (clinical trial number: not applicable).

### Phase I: screening for cardiovascular disease risk

#### Sampling method

Convenience sampling method was applied.

##### Sample size

The sample size was calculated using the PASS 11 program (version 11.0.8), with a confidence level of 99% and a margin of error of 0.05. After reviewing previous study results in *Egypt STEPS Survey* [4] which showed that the prevalence of CVDs risk greater than 30% in the Egyptian population was 7.7%, a sample size of at least 210 administrative staff was found suitable to achieve the study objectives.

##### Data collection tool

For phase I, a self-administered structured questionnaire was used to gather data between January and March 2023 [18] (translated into Arabic by a bilingual expert and underwent back-translation by another bilingual expert to ensure the accuracy of the translation). The questionnaire was composed of four sections. Section I: Socio-demographic characteristics and special habits of the studied participants, including name and telephone number, age, gender, education, marital status, and residence. Section II: A family history of CVDs, limited to first-degree relatives, smoking, physical inactivity, diabetes, hypercholesterolemia, and hypertension, are lifestyle factors linked to an increased risk of CVDs. Section III Using standardized protocols and calibrated equipment, anthropometric measures and blood pressure readings were obtained from each participant. (Weight, height, hip circumference (HC), waist circumference (WC), body mass index (BMI), and waist-to-hip ratio (WHR). Section IV: The WHO risk prediction chart for non-laboratory-based CVDs. It calculates the estimated risk of CVDs: including BMI, systolic blood pressure, smoking status, age, and gender. Based on their results, participants were then divided into three risk categories: low (less than 10%), moderate (10–20%), and high (more than 20%) [11]. The WHO CVDs risk assessment has been validated in two regions: sub-Saharan Africa and the Eastern Mediterranean. By taking into consideration regional differences in risk variables and health profiles, this guarantees that the evaluation appropriately forecasts cardiovascular risk across a variety of populations [11].

### Phase II: Quasi experimental study (Intervention by educational program)

#### Sampling method

Convenience sampling was also applied in this phase.

##### Sample size

**Fig. 1:**
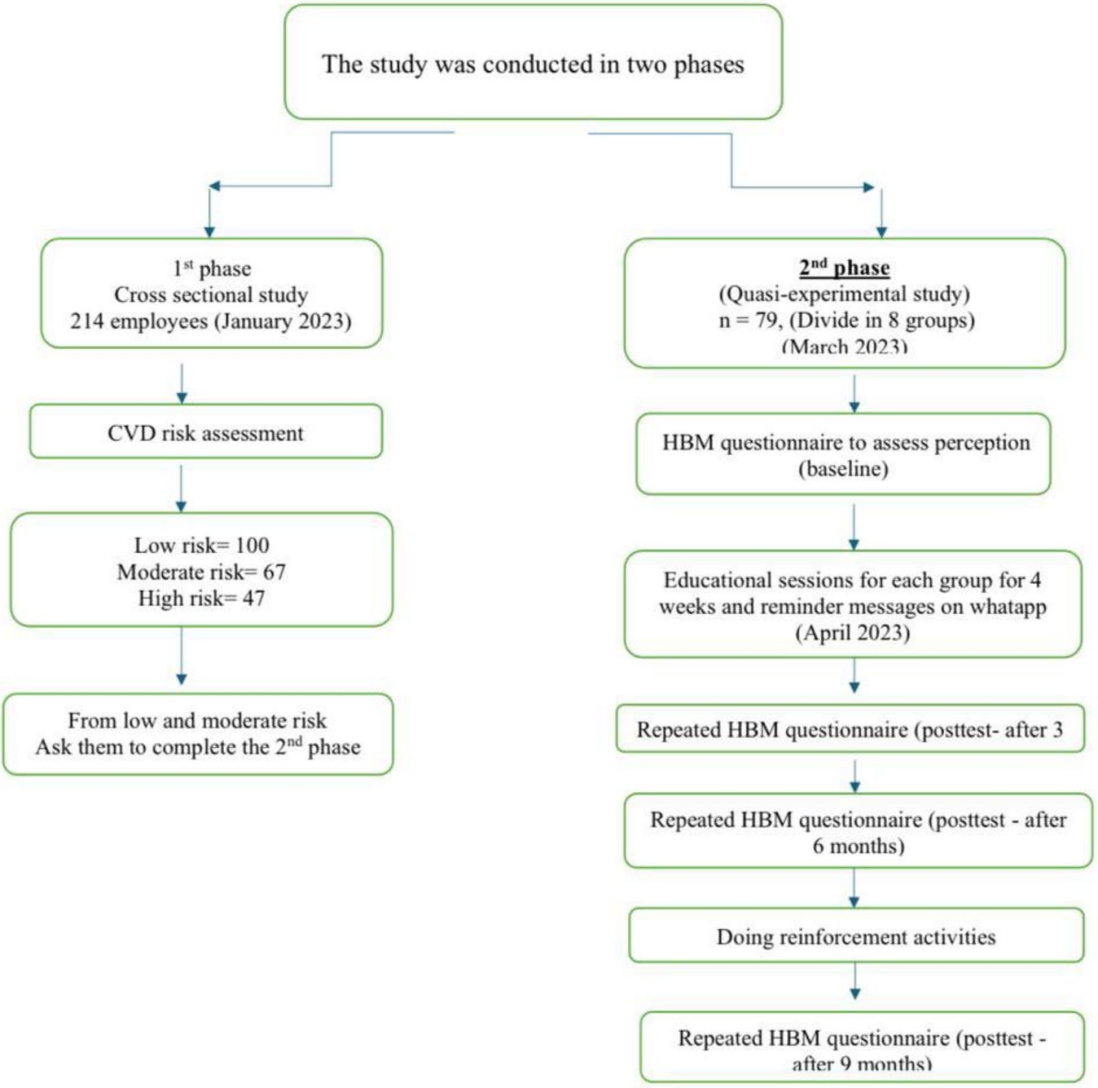
A study design for the participants over the two phases

Using the PASS 11 program (version 11.0.8), The following criteria were used to determine the sample size for before-and-after research: α (two-tailed) = 0.05; β = 0.20, based on results from the previous study by *Saffari *et al [19].*,* which showed that the mean of perceived severity domain score as one of HBM domains before and after the intervention was 3.01 ± 0.32 and 3.36 ± 0.24, respectively, with an effect size = 1.23. Thus, a sample size of at least 60 workers at risk for cardiovascular disease would be sufficient to achieve the objectives of this phase. The perceived severity' domain was selected as it was identified as the most crucial element for influencing participant behavior. Out of 167 individuals eligible to participate in the second phase—according to their CVDs risk assessment status—79 agreed to participate in the second phase (high risk individuals were excluded and advised to seek medical advice). Accordingly, almost 50% of respondents (47.3%) agreed to participate in the second phase. All of these 79 employees completed all stages of the quasi experiment, leading to 100% participation and retention rates at every follow-up point. This reduced the possibility of selection bias and supports the generalizability of the findings within the study context) (the diagram of the study design is shown in Fig. 1).

##### Data collection tool

Data was collected between May 2023 and February 2024. A self-administered structured questionnaire was modified from the “Knowledge of CVDs” and the “HBM scale for CVDs.” [18, 19]. A bilingual expert translated the questionnaire into Arabic, and another bilingual expert translated it back into English. A pilot test was also carried out on 10% of the total sample (*n* = 21) to determine the time required to complete the questionnaire and to evaluate the ease of use, clarity, feasibility, and applicability of a self-administered questionnaire and HBM scale. Nothing was changed after the pilot test.

Thus, the survey was divided into two section.

##### Section I

Knowledge about cardiovascular risk factors (Amdemariam et al., 2022). There were ten questions about knowledge, to which the options were "yes" or "no." Participants who provided accurate answers to 50% or more of the questions regarding CVDs prevention were considered to have good knowledge. Respondents with inadequate knowledge were defined as those who could not correctly answer more than 50% of the questions regarding CVDs prevention. The score of this scale was compared at baseline and 3 months and also was the change in knowledge among the participants.

##### Section II

The CVDs HBM. It consists of seven domains each on a five level Likert scale: “Perceived susceptibility” (four items), perceived severity (five items), "perceived benefits" (eight items), “perceived barriers” (six items), “cues to action” (three items), “self-efficacy” (six items), and “preventative behavior” (five items) with a total of 37 items. Prior research evaluating the HBM scale's test–retest reliability found a correlation coefficient of r = 0.88, which indicates strong stability over time. Regarding Cronbach's alpha, the internal consistency was 0.77, indicating a satisfactory level of reliability [20].

Method of scoring: The range of possible total scores lies between 37 and 148. A series of item responses, from completely disagree (1) to completely agree (4), is used in HBM. Better behaviors, attitudes, and awareness are correlated with higher scores. On the barrier subscale, however, higher values correspond to more negative perspectives on health [19].

##### Phases of the experimental study

The study was composed of three stages: pre-intervention stage, intervention phase and post-intervention phase.

1. Pre-intervention stage*:* In order to evaluate the perceptions of the administrative staff under study, baseline data were gathered and examined during this program phase. The questionnaire took an average of 15 to 20 min to complete.
2. Intervention stage: To improve engagement and comprehension, the researchers employed a variety of teaching techniques, such as group discussions, visual aids, printed materials, presentations, and Arabic brochures, thus providing a comprehensive educational intervention that addressed the target group's knowledge gaps regarding CVDs risk factors. An emeritus professor specialized in behavioral medicine and two senior scholars revised the health education material. Multiple measures were implemented to ensure fidelity to the intervention. The initial step was to use the same intervention materials for every participant. Second, we repeated the evaluation three times at three-month intervals, so delivery was ensured by periodic supervision. Also adherence was recorded using checklists that prime researcher filled out at the end of each session. Even more, all health education was provided by the same researcher, the prime researcher. The administrative staff members were divided into eight groups of ten, and a list with each participant's mobile number was compiled. A WhatsApp group including the participants and the prime researcher was then set up. The researcher scheduled a total of 24 sessions at times that were both convenient for the participants. Each group was given three sessions, with two sessions held per day based on the time preferences and availability of the participants. Each of the three face-to-face training sessions lasted from 60–90 min allowing time for interaction with the researcher (based on the recommended duration HE sessions [21]). The sessions were conducted weekly over three weeks for each group and were held in the participants’offices to ensure convenience and comfort. The first session discussed the prevalence and seriousness of CVDs in Egypt, as well as the statistics, complications, and negative consequences linked to risk factors and poor health behaviours that participants might face. The second session focused on preventative therapy and lifestyle modifications, discussing healthy behaviors like diet and exercise and potential challenges that might be faced in implementing these behaviors. The third session was an open discussion based on questions from the HBM for CVDs, emphasizing self-efficacy, social support, and environmental factors in influencing health behaviors. Educational messages were also reinforced through online messaging and pamphlets were distributed after the training sessions.
3. Post-intervention stage: The researchers assessed administrative staff' perceptions and preventive behavior after the training sessions using post-tests to measure HBM-related constructs at 3-month, 6-month and 9-month follow-up periods. Blood pressure and anthropometric measures were only reassessed at 9 months. These measurements included weight, BMI, WHR, and blood pressure to evaluate any long-term physical health outcomes from the training. Knowledge about CVDs risk was reassessed after 3 months. During the period of follow-up, the prime researcher continuously checked the WhatsApp group, responding to any queries and providing participants with essential instructions. WhatsApp messages were sent twice a week throughout the follow up period.

### Statistical analysis

The data collected was coded, checked for accuracy, and entered into a personal computer. IBM SPSS (Statistical Package for Social Science) software version 24.0 was used for all data processing and statistical analyses. Frequencies and percentages were used to report qualitative category variables. Quantitative variables were presented as means with the standard deviation (SD). One-way analysis of variance (ANOVA) was used to compare more than two groups, and the Bonferroni post hoc comparison test was performed. Pearson's Chi-square was used to compare the qualitative variables. Mauchly’s test was not significant, so sphericity was assumed. RM ANOVA was significant, so post-hoc pairwise comparison was conducted to identify where the differences lie between the levels of the repeated measure. HBM subscales and total scores were computed. The level of significance was set at *p* < 0.05 for this study.

### Ethical consideration

The Research Ethical Committee (REC) at the ASU Faculty of Medicine provided its approval (with code FWA 000017585 FAMSU MD 149/2022). informed consent was obtained after each participant was told of the study's goals and phases, and the faculty of medicine obtained administrative approval. The information was collected with the utmost confidentially and used only for research.

## Results

As shown in Table 1, the studied sample had a mean age of 48.9 ± 7.95 years, with 74.3% of the participants being female. The majority of them were married (90.2%), 90.7% had a college degree, 86.7% lived in an urban area, 3.7% of people were smokers, 69.6% had a sedentary lifestyle, 18.7% of people with diabetes mellitus, 30.8% with hypertension, 16.4% with hypercholesteremia, 21.5% with a family history of CVDs, 65.9% with obesity, and 53% with a high-risk category of WHR.

**Table 1:** Sociodemographic data and medical history among the studied sample (*n* = 214)

Variables		n	%
**Sociodemographic characteristics**			
**Age in years**	**Mean ± SD (min–max)**	48.9 ± 7.95(40–66)	
**Gender**	Male	55	25.7%
	Female	159	74.3%
**Marital status**	Single	13	6.1%
	Married	193	90.2%
	Divorced/Widow	8	3.7%
**Educational level**	Graduated from secondary school	9	4.2%
	Bachelor	194	90.7%
	Master	11	5.1%
**Residence**	Urban	190	88.7%
	Rural	24	11.3%
**Weekly working hours. Mean ± SD (min–max)** 43.13 ± 10.09 (24–84)			
**Special habits & Medical history**		n	%
**Smoking**	Yes	8	3.7%
	No	206	96.3%
**Regular physical activity**	Yes	65	30.4%
	No	149	69.6%
**Having Diabetes**	Yes	40	18.7%
	No	174	81.3%
**Having Hypertension**	Yes	66	30.8%
	No	148	69.2%
**Having Hypercholesteremia**	Yes	35	16.4%
	No	179	83.6%
**Family history of CVD**	Yes	46	21.5%
	No	168	78.5%
**Anthropometric measurements**			
**Body mass index (BMI)**	Normal	15	7%
	Overweight	58	27.1%
	Obese	141	65.9%
**Waist/hip ratio (WHR)**	Low	71	33.2%
	Moderate	68	31.8%
	High	75	35%

As shown in Table 2 According to the WHO CVDs risk assessment,47 (22%) were at high risk, 67 (31.3%) were at moderate risk, and 100 (46.7%) were at low risk. When comparing the WHO CVDs risk prediction chart between males and females, there was a significant difference. Most males had a moderate to high risk of developing CVDs. Males mainly had a moderate and high risk to develop CVDs.

**Table 2:** Assessment of CVD risk according to the WHO/CVD risk chart(*n* = 214)

CVD risk assessment	Total		Male		Female		Effect size	*P* value
	n	%	n	%	n	%		
Low risk (< 10%)	100	(46.7%)	12	(21.8%)	88	(55.3%)	0.305	< 0.001
Moderate (10–20%)	67	(31.3%)	28	(50.9%)	39	(24.5%)		
High risk (> 20%)	47	(22%)	15	(27.3%)	32	(20.1%)		

(CVD risk assessment includes age, gender, smoking status, SBP, and BMI)

As shown in Table 3 The “perceived susceptibility domain score to CVDs” was not associated with any of the risk factors, except for Waist-to-Hip Ratio (WHR), where higher WHR was associated with a greater “perceived susceptibility” score. There was a significant association between mean “perceived severity domain score and certain risk factors such as: age, being free from hypercholesterolemia, and a positive family history (FH) of CVDs. Participants aged 40– < 50 had higher “perceived severity domain score compared to other age groups. Higher “perceived benefits” domain score was significantly associated with being aged 40– < 50, male sex, being married, being well-educated, and being free from hypercholesterolemia.

**Table 3:** Factors affecting the scores of the different domains of the health belief model for CVD scale (*n*=79)

Variables		Perceived susceptibility Min–max (4–16)	Perceived severity Min–max (5–20)	Perceived benefits Min–max (8–32)	Perceived barriers Min–max (6–24)
Age	40- < 50	7.5 ± 1.8	9.1 ± 2.4	15.8 ± 4.2	15.2 ± 4.3
	50–60	7.9 ± 1.7	8 ± 2.5	13.5 ± 3.5	16 ± 3.5
	> 60	7.7 ± 1.3	6.9 ± 2.6	12.9 ± 3.3	17.1 ± 2.5
	*P* value	0.738	0.041*	0.039*	0.277
Gender	male	7.7 ± 1.8	9 ± 2.6	16.2 ± 4	15.8 ± 4.3
	female	7.8 ± 1.6	7.7 ± 2.6	13.2 ± 3.4	16.1 ± 3.3
	*P* value	0.976	0.063	0.002*	0.742
Educational level	diploma	8.6 ± 1.7	6.4 ± 3.1	9.6 ± 2.6	16.6 ± 2
	bachelor	7.7 ± 1.6	8.2 ± 2.5	14.2 ± 3.7	16 ± 3.6
	*P* value	0.231	0.147	0.007*	0.711
Marital status #	Single	7.5 ± 2.1	10 ± 4.2	12 ± 2.8	16 ± 0
	Married	7.8 ± 1.6	8.2 ± 2.5	14.3 ± 3.7	15.9 ± 3.6
	Divorced/widow	7.8 ± 1.8	5.6 ± 2.4	9.8 ± 2.5	18.6 ± 1.7
	P value	0.975	0.056	0.026*	0.248
Suffering from hypertension	yes	8.1 ± 1.5	7.9 ± 2.7	14.1 ± 4.1	16.5 ± 3.7
	no	7.5 ± 1.7	8.1 ± 2.6	13.8 ± 3.6	15.7 ± 3.5
	*P* value	0.114	0.711	0.787	0.302
Suffering from Diabetes mellitus	Yes	8.2 ± 1.7	7.9 ± 2.4	14.3 ± 3.5	16.8 ± 3.2
	No	7.5 ± 1.6	8.1 ± 2.7	13.7 ± 3.9	15.6 ± 3.7
	*P* value	0.059	0.715	0.491	0.181
Suffering from hypercholesteremia	yes	7.6 ± 1.8	6.8 ± 2.3	12.1 ± 3.5	16.2 ± 3.9
	no	7.8 ± 1.6	8.4 ± 2.6	14.5 ± 3.7	16 ± 3.5
	*P* value	0.727	0.015*	0.012*	0.853
FH of CVD	yes	8.7 ± 1.6	6.2 ± 2.1	12.8 ± 3.2	15.1 ± 4.3
	no	7.6 ± 1.6	8.3 ± 2.6	14.1 ± 3.8	16.1 ± 3.5
	*P* value	0.073	0.025*	0.335	0.416
Waist/hip ratio#	normal	7.1 ± 1.6	8.3 ± 2.6	13.1 ± 4	16.4 ± 2.7
	moderate	8.1 ± 1.6	8.3 ± 2.1	14.4 ± 3	16.6 ± 3.4
	high	8.2 ± 1.5	7.5 ± 3	14.4 ± 4.1	15.2 ± 4.5
	*P* value	0.013*	0.440	0.348	0.309
BMI#	Normal	7.1 ± 1.5	8.5 ± 2.8	14.4 ± 4.1	15.8 ± 3.5
	Overweight	7.8 ± 1.8	7.8 ± 2.7	13.9 ± 3.7	16.3 ± 3.7
	Obese	8 ± 1.5	8 ± 2.5	13.8 ± 3.8	15.8 ± 3.6
	*P* value	0.215	0.695	0.866	0.810

(*) *P* value ≤ 0.05 is considered statistically significant^.^ Independent t test was used. (#) Anova test was used

Furthermore “cues to action domain” score was statistically associated with having Diabetes Mellitus, and higher “self-efficacy domain” score was statistically associated with having hypercholesterolemia. There were no significant associations between sociodemographic factors and perceived barriers domain score or preventive behavior domain score. (see supplementary Table 1).

Table 4 demonstrates that the intervention produced significant improvements across nearly all Health Belief Model domains, with the most notable effect observed in knowledge (large effect size = 1.654, *p* < 0.001). Moderate improvements were seen in perceived barriers (0.312), total health score (0.322), self-efficacy (0.257), and perceived benefits (0.279), while smaller yet significant effects were observed for perceived severity (0.198), susceptibility (0.141), and cues to action (0.130). In contrast, preventive behavior showed no significant change (effect size = 0.010, *p* = 0.390), indicating a gap between improved perceptions and actual behavioral practice. There is a statistically significant change between measurements before and after 9 months of HBM application, except for blood pressure measurement.

**Table 4:** A comparison between the domains of the health belief model across 0, 3, 6, and 9 months following the intervention (*n* = 79)

Variables	Baseline	After 3 months	After 6 months	After 9 months	Effect size	P value
Knowledge ^(&)^	6.05 ± 1.78	9.31 ± 0.85			1.654	< 0.001*
Perceived susceptibility	7.74 ± 1.63	9.49 ± 2.95	8.79 ± 1.96	8.34 ± 1.70	0.141	**0.001**
Perceived severity	8.03 ± 2.59	9.83 ± 2.21	9.17 ± 2.38	8.87 ± 2.11	0.198	**0.001**
Perceived benefits	13.92 ± 3.76	17.62 ± 3.37	15.82 ± 3.47	15.02 ± 3.91	0.279	**0.001**
Perceived barriers	16.02 ± 3.55	12.49 ± 4.01	13.54 ± 3.81	14.13 ± 3.09	0.312	**0.001**
Cues to action	4.70 ± 1.87	5.79 ± 1.73	5.32 ± 1.54	5.00 ± 1.85	0.130	**0.001**
Self-efficacy	10.12 ± 3.61	13.45 ± 2.89	12.00 ± 2.01	11.26 ± 3.24	0.257	**0.001**
Preventive behavior	7.62 ± 2.91	7.98 ± 2.44	7.65 ± 2.48	7.64 ± 2.75	0.010	0.390
Total health score	68.18 ± 6.94	76.68 ± 6.13	72.32 ± 6.71	70.29 ± 7.05	0.322	**0.001**

^(*) ^^*P* value ≤0.05 is considered statistically significant #repeated measure ANOVA was used. (&) paired t test was used^

Table 5 shows how HBM subscales and preventative behavior changed from baseline to follow-up. Significant improvements were observed in all subscales, except for “preventive behavior" domain score, from baseline to the 3-month follow-up. However, there was a slight decline in these outcomes at the 6- and 9-month follow-up periods.

**Table 5:** Mean distribution of anthropometric and blood pressure measurements of the studied sample before and after health belief model application (*n* = 79)

**Variables**	**Baseline**	**After 9 months**	Effect size	***P* value**
	Mean ± SD			
Waist/hip ratio	0.87 ± 0.06	0.86 ± 0.06	0.015	0.001*
Body mass index	33.97 ± 5.53	33.23 ± 5.07	1.190	0.001*
Systolic blood pressure	124.75 ± 9.44	124.11 ± 8.83	2.927	0.058
Diastolic blood pressure	81.71 ± 10.5	81.32 ± 10.12	1.923	0.083

^(*) ^^*P* value ≤0.05 is considered statistically significant #paired t test was used^

## Discussion

The present study investigated how a lifestyle modification program based on HBM can modify CVDs risk factors. Out of the 214 administrative staff who participated in the current study, with a mean age of 48.9 ± 7.95 years, 74.3% were female. Based on the WHO/ISH prediction risk score, 46.7% had a low risk, 31.3% had a moderate risk, and 22% had a high risk of CVDs for the following 10 years.

These results are comparable to those of a previous study by Awad A. MB. and Khalil WA. (2021) [17], which used the Framingham Risk Score to assess the administrative staff at Zagazig University in Egypt and discovered that 42% of participants had low risk, 30% had moderate risk, and 28% had high risk for CVDs. Babatunde et al. (2020) [22] used the WHO/CVDs risk score in southwest Nigeria and discovered that 14.6% had a high risk of CVDs, 8.5% had a moderate risk, and 76.9% had a low risk. The change in CVD risk levels is probably explained by the significant difference in obesity rates between this sample and the current study (24.6% vs. 65.9%). Obesity is a known risk factor for cardiovascular illnesses; therefore, the increased incidence of obesity in this study would have explained why there were more people at moderate to high risk for CVDs.

This study also revealed significant difference in CVDs risk stratification between males and females (*p* < 0.001). Males had a higher risk of developing CVDs especially for moderate and high-risk categories. *Ghorpade *et al*. (2015)* [23] found similar results among rural populations in the South-East Asian region, using the WHO/ISH risk prediction charts (*p* = 0.017).

Regarding factors affecting the behavioral intention and barriers of administrative staff based on HBM scale, our study revealed that “perceived severity” domain showed a statistically significant association with age Furthermore, as compared to other age groups, administrative workers in the 40–50 age range scored higher in the "perceived severity" domain. This result aligns with the findings of Alshaikh M. (2019) [24]. A greater understanding of the health hazards of ageing and the long-term effects of lifestyle choices may be the cause of this age group's elevated perception of severity. Additionally, people with a family history of CVDs reported perceived severity to be considerably higher.

The current study identified a statistically significant association between “perceived benefits” domain score and several demographic factors, including age, gender, marital status and different educational levels. Similarly, the findings of *Alshaikh M. (2019)* [24] also showed *a* significant association between the “perceived benefits” domain score and marital status and educational level.

Karimy et al. (2016) [25] highlighted the influence of education on health perceptions by reporting a significant association between all HBM subscales and different educational degrees. This could be explained by the fact that higher educational levels are linked to a better understanding of health risks, greater recognition of the benefits of preventive behaviors, and enhanced confidence in one's ability to take action (self-efficacy) [26].

After the educational program, awareness of risk factors of CVDs significantly improved. This is in line with the quasi-experimental study conducted by *Kheiri *et al., (2019) [27] in Iran, which reported a significant change in awareness scores among their participants. This is also in line with findings of *Midjani, Hossaini and Sharifi, (2023)* [28] whose study revealed a significant change in “awareness” following the intervention.

At three months, all HBM domain scores had changed significantly, with the exception of the "preventive behaviour" domain, in terms of the follow-up time. A minor improvement was observed in this domain over time, although it was not statistically significant. Nonetheless, the minor decline in the other domains throughout the six- and nine-month follow-up highlights the challenges in maintaining behavioural modifications over an extended period. Similar studies with follow-up periods longer than three months were not found.

The attenuation of effects seen at 6 to 9 months may be due to reduced participant motivation over time, difficulties maintaining lifestyle changes without continuous support, and the impact of environmental or social factors that inhibit sustained behavioral change. Future initiatives might include regular booster sessions to reinforce important ideas, offer continuous involvement via social media or mobile health platforms, and create peer support networks to improve long-term impact. These approaches could support the ongoing adoption of healthy behaviors, encourage accountability, and keep participants motivated.

Similar results were reported by Fatahian et al. (2024) [29] in Iran, who assessed patients after one and three months after a cardiovascular event. They studied the effects of an HBM-based educational intervention on patients' eating patterns following myocardial infarction. The results showed that the intervention group's mean scores on HBM subscales differed significantly between the three times, except for the perceived barrier subscale, which increased after one month and then slightly declined after three months.

The current study found a significant increase in “*perceived susceptibility” and “perceived severity”* domain score three months post-intervention. This is consistent with the findings of *Kheiri *et al., (2019) [27]* -*who conducted a quasi-experimental study -, *Midjani, Hossaini and Sharifi, (2023) *[28], as well as *Mohammadkhah *et al*.(2023)* [30] among hypertensive patients. All these studies were carried out in Iran.

*Ammouri *et al*. (2011)* [31] reported that people with low perceived susceptibility domain score regarding the risks of CVDs tend to have lower adherence to healthy dietary behaviors. This low perception negatively influences the cost–benefit evaluation of adopting a behavior and the resulting decisions. Therefore, identifying the perceived susceptibility and severity domain score of a disease and its enhancement by expressing negative outcomes associated with the disease will help improve preventive behaviors.

Regarding *“perceived benefits” and “perceived barriers”* domain score*,* the current study revealed a significant increase in the mean score three months post-intervention. This finding is consistent with the study by *Chatripour *et al*. (2016)* [32]*,* who conducted quasi-experimental study in Iran. Other studies supporting these findings include studies by *Kheiri *et al*. (2019)* [27]*; Saffari *et al*. (2020)* [19]*; MohammedKhah *et al*. (2023)* [30].

The findings of the current study indicate a statistically significant rise in the values of *the “cues to action”* domain score which is consistent with the results of, *Chatripour *et al*., (2016)* [32]*; Kheiri *et al*. (2019)* [27]*;* and *MohammedKhah *et al*. (2023)* [30]. On the other hand, Saffari et al. (2020)19 found that the mean score of the "cues to action" category had not changed significantly. The difference between the significant changes in our study and the small changes in the mean "perceived severity" and "perceived susceptibility" domain scores in the Saffari et al. (2020) [19] study could be the cause of this discrepancy. These differences highlight the potential impact of “perceived severity” and “perceived susceptibility” domain score on the effectiveness of cues to action, suggesting that addressing these factors could enhance intervention outcomes***.***

The findings show a notable increase in the "self-efficacy" domain score, which is in line with findings from MohammedKhah et al. (2023) [30]; Saffari et al. (2020) [19]; and Chatripour et al. (2016) [32]. It is recommended to use strategies including emphasizing real-world accomplishments, offering incentives, using role models, and creating alternative experiences in order to further increase self-efficacy. Setting short-term, attainable goals might also help people feel more confident in their skills.

*Regarding* “*preventive behavior" domain score,*while the total score did not show significant improvement (*p* = 0.390), most individual items of the domain exhibited significant changes, except item " I do not consume fast foods and fried/fatty foods regularly" It is noteworthy that after removing this question from the analysis, significant differences emerged. The lack of significant change in dietary habits related to fast foods suggests that this behavior is more resistant to change. Fried foods are a staple in Egyptian cuisine, playing a prominent role in daily meals and social gatherings. This cultural reliance on fried foods presents a significant challenge for health promotion initiatives aimed at reducing unhealthy dietary practices [33]. This highlights the influence that specific dietary habits can have on overall health behavior evaluations.

Regarding Anthropometric measurements before and after the educational program based on HBM, the current study found a slight but significant decrease in BMI and waist-to-hip ratio (WHR) following the educational program. This finding is consistent with the study by *Saffari *et al*. (2020)* [19] in Iran, which reported a slight but significant decrease in BMI before and after their educational intervention. Changes in BMI and WHR require a consistent and sustained approach to diet, exercise, and overall lifestyle changes. Tracking these metrics over a longer period would allow for a clearer understanding of long-term progress and health outcomes.

There was no statistically significant change in blood pressure measurements according to the current study. Similarly, previous studies conducted by *Saffari *et al*.(2020)* [19] in Iran and *Karmakar *et al*.(2023)* [34] in Kolkata, India didn’t find any change in blood pressure measurements. However, a quasi-experimental study carried out in Shiraz, Iran, by *Mohammadkhah *et al*.(2023)* [30] revealed significant differences in the experimental group's blood pressure measurement before and after the program. Their study was carried out specifically on hypertensive patients. Thus, this disparity could arise from the differences in the study populations and the fact that their educational sessions focused on hypertension and its related effects.

The HBM is an easy tool that can be used as a foundation for national and individual health education. Including the HBM in public health campaigns could increase medical professionals' and people's awareness of the modifiable risk factors for CVDs. People can acquire healthier habits and have a better awareness of the personal significance of risk reduction by using the HBM as a framework for health education [35].

Additionally, workplaces can play a critical role in supporting cardiovascular health. Employers can promote a better lifestyle by doing things like offering wholesome food options, creating spaces for exercise, and hosting regular, easily accessible health education seminars for administrative staff. The Ministry of Health can enhance its preventative efforts by integrating the HBM into national education programs. This approach can boost the effectiveness of health messaging, motivate individuals to take preventative action, and support healthcare professionals in delivering behavior-focused interventions.

### Study limitations

The study lacks a comparison group because it is a quasi-experiment. To overcome this constraint, a pretest–posttest strategy with repeated measurements at 3, 6, and 9 months was implemented. This design was also chosen in order to address compliance issues and to avoid small subgroup sizes. This design also allows better participant engagement. The generalizations to a larger population are not possible because the participants were limited to one institution. Future studies could be strengthened by applying more rigorous designs, like cluster-randomized trials, which offer better causal inference while still being feasible in community or workplace contexts.

## Conclusions

According to their WHO/ISH prediction risk score, over the half of the participants were at moderate to high risk for CVDs. Also, this study found that a relatively short educational program based on the HBM effectively raised the administrative staff' awareness of behavioral risk factors for CVDs. All HBM domain scores showed a significant increase at the three months evaluation, except for “preventive behavior” domain score. However, evaluation at six months and nine months, showed a small decline in the HBM domain scores, indicating an area that needs more investigation.

## Supplementary Information

Supplementary Material 1.

## Data Availability

No datasets were generated or analysed during the current study.

## Abbreviations

CVDs: Cardiovascular diseases
HBM: Health belief model
ASU: Ain Shams University
PA: Physical activity
QRISK: QRESEARCH cardiovascular risk algorithm
SCORE: Systematic Coronary Risk Evaluation
WHO: World Health Organization (WHO)/ISH chart
FRS: Framingham Risk Score
TPB: Theory of Planned Behavior
SCT: Social Cognitive Theory
BMI: Body mass index
WC: Waist circumference
HC: Hip circumference
WHR: Waist-hip ratio
SPSS: Statistical Package for Social Science
SD: Standard deviation
ANOVA: One-way analysis of variance
RMNOVA: Repeated measure ANOVA
REC: Research Ethical Committee
