# Supplementary material for: Assessing the impact of educational intervention based on a health belief model to modify cardiovascular disease risk factors among Egyptian University administrative staff: a quasi-experimental study
Source: BMC Cardiovasc Disord. 2025 Dec 5;25:865. doi: 10.1186/s12872-025-05359-3 (PMC12699899; doi:10.1186/s12872-025-05359-3)
Supplement: Supplementary file 1 — Supplementary Material 1. [file 12872_2025_5359_MOESM1_ESM.docx]

**Table (1): Factors affecting the scores of the different domains of the health belief model for CVD scale (n=79):**

| Variables | | Perceived susceptibility  Min-max  (4-16) | Perceived severity  Min-max  (5-20) | Perceived benefits  Min-max  (8-32) | Perceived barriers  Min-max  (6-24) | Cues to action  Min-max  (1-9) | Self-efficacy  Min-max  (2-18) | Preventive behavior  Min-max  (3-14) |
| --- | --- | --- | --- | --- | --- | --- | --- | --- |
| Age | 40-<50 | 7.5±1.8 | 9.1±2.4 | 15.8±4.2 | 15.2±4.3 | 4.84±1.8 | 8.74±3.38 | 7.32±2.14 |
|  | 50-60 | 7.9±1.7 | 8±2.5 | 13.5±3.5 | 16±3.5 | 4.44±1.83 | 10.31±3.67 | 7.42±3 |
|  | >60 | 7.7±1.3 | 6.9±2.6 | 12.9±3.3 | 17.1±2.5 | 5.33±2.06 | 11.33±3.37 | 8.6±3.42 |
|  | P value | 0.738 | 0.041* | 0.039* | 0.277 | 0.268 | 0.099 | 0.352 |
| Gender | male | 7.7±1.8 | 9±2.6 | 16.2±4 | 15.8±4.3 | 4.79±1.96 | 10.11±3.26 | 6.74±1.91 |
|  | female | 7.8±1.6 | 7.7±2.6 | 13.2±3.4 | 16.1±3.3 | 4.68±1.86 | 10.13±3.74 | 7.9±3.12 |
|  | P value | 0.976 | 0.063 | 0.002* | 0.742 | 0.837 | 0.975 | 0.130 |
| Educational level | diploma | 8.6±1.7 | 6.4±3.1 | 9.6±2.6 | 16.6±2 | 6.2±2.68 | 7.4±5.5 | 7.4±3.78 |
|  | bachelor | 7.7±1.6 | 8.2±2.5 | 14.2±3.7 | 16±3.6 | 4.61±1.79 | 10.31±3.42 | 7.64±2.87 |
|  | P value | 0.231 | 0.147 | 0.007* | 0.711 | 0.066 | 0.081 | 0.863 |
| Marital status # | Single | 7.5±2.1 | 10±4.2 | 12±2.8 | 16±0 | 6±2.08 | 11.71±1.41 | 8±3.41 |
|  | Married | 7.8±1.6 | 8.2±2.5 | 14.3±3.7 | 15.9±3.6 | 4.49±1.76 | 9.83±3.71 | 7.41±2.82 |
|  | Divorced/widow | 7.8±1.8 | 5.6±2.4 | 9.8±2.5 | 18.6±1.7 | 5.80±2.28 | 11.80±2.86 | 9.80±3.03 |
|  | P value | 0.975 | 0.056 | 0.026* | 0.248 | 0.050 | 0.242 | 0.199 |
| Suffering from hypertension | yes | 8.1±1.5 | 7.9±2.7 | 14.1±4.1 | 16.5±3.7 | 4.58±1.64 | 9.91±3.96 | 7.64±3.07 |
|  | no | 7.5±1.7 | 8.1±2.6 | 13.8±3.6 | 15.7±3.5 | 4.8±2.04 | 10.28±3.37 | 7.61±2.82 |
|  | P value | 0.114 | 0.711 | 0.787 | 0.302 | 0.583 | 0.662 | 0.968 |
| Suffering from Diabetes mellitus | Yes | 8.2±1.7 | 7.9±2.4 | 14.3±3.5 | 16.8±3.2 | 4.18±1.52 | 9.75±4.06 | 8.32±2.63 |
|  | No | 7.5±1.6 | 8.1±2.7 | 13.7±3.9 | 15.6±3.7 | 5±2 | 10.33±3.36 | 7.24±3.01 |
|  | P value | 0.059 | 0.715 | 0.491 | 0.181 | 0.044* | 0.520 | 0.100 |
| Suffering from hypercholesteremia | yes | 7.6±1.8 | 6.8±2.3 | 12.1±3.5 | 16.2±3.9 | 4.74±2.23 | 11.47±2.7 | 7.63±3.18 |
|  | no | 7.8±1.6 | 8.4±2.6 | 14.5±3.7 | 16±3.5 | 4.7±1.77 | 9.7±3.77 | 7.62±2.85 |
|  | P value | 0.727 | 0.015* | 0.012* | 0.853 | 0.948 | 0.029* | 0.986 |
| FH of CVD | yes | 8.7±1.6 | 6.2±2.1 | 12.8±3.2 | 15.1±4.3 | 4.84±1.8 | 8.74±3.38 | 7.32±2.14 |
|  | no | 7.6±1.6 | 8.3±2.6 | 14.1±3.8 | 16.1±3.5 | 4.44±1.83 | 10.31±3.67 | 7.32±2.14 |
|  | P value | 0.073 | 0.025* | 0.335 | 0.416 | 0.928 | 0.285 | 0.965 |
| Waist/hip ratio# | normal | 7.1±1.6 | 8.3±2.6 | 13.1±4 | 16.4±2.7 | 4.90±2.05 | 10.43±3.10 | 7±2.66 |
|  | moderate | 8.1±1.6 | 8.3±2.1 | 14.4±3 | 16.6±3.4 | 4.47±1.47 | 9.52±3.88 | 7.34±2.93 |
|  | high | 8.2±1.5 | 7.5±3 | 14.4±4.1 | 15.2±4.5 | 4.69±2.01 | 10.30±3.96 | 8.57±3.02 |
|  | P value | 0.013* | 0.440 | 0.348 | 0.309 | 0.724 | 0.635 | 0.112 |
| BMI# | Normal | 7.1±1.5 | 8.5±2.8 | 14.4±4.1 | 15.8±3.5 | 4.75±2.54 | 10.75±2.23 | 6.75±1.80 |
|  | Overweight | 7.8±1.8 | 7.8±2.7 | 13.9±3.7 | 16.3±3.7 | 4.54±1.56 | 10.48±3.67 | 8.06±3.34 |
|  | Obese | 8±1.5 | 8±2.5 | 13.8±3.8 | 15.8±3.6 | 4.86±1.83 | 9.40±4.08 | 7.60±2.84 |
|  | P value | 0.215 | 0.695 | 0.866 | 0.810 | 0.795 | 0.370 | 0.339 |

^(*)P value ≤0.05 is considered statistically significant (&) t-test (#)ANOVA^

**Figure (1) : Change of HBM domains over follow up period 0, 3,6 and 9 months (n = 79).**

**^NB:^** ^For the five subscales, higher scores indicate extremely healthy beliefs. However, for the subscale concerning barriers, higher scores indicate more negative health beliefs.^
